# Alarming Surge in Early-onset Type 2 Diabetes: A Global Catastrophe on the Horizon

**DOI:** 10.17925/EE.2023.19.2.5

**Published:** 2023-07-13

**Authors:** Arefin Sadat

**Affiliations:** Chandpur Medical College, Chandpur, Bangladesh

**Keywords:** Autoimmunity, community health, insulin-dependent diabetes, preventive medicine, type 2 diabetes, youth-onset type 2 diabetes

## Abstract

Diabetes poses a significant threat to public health. In the last 30 years, the worldwide incidence of type 2 diabetes mellitus (T2DM) has increased drastically among adolescents. Since the number of young people with T2DM is rising, it is anticipated that early-onset T2DM will become a common characteristic of the diabetes population in developed and developing nations. Current evidence suggests that β-cell function declines more rapidly in early-onset T2DM than in older-onset T2DM. In addition, early-onset T2DM appears to be associated with a greater risk of complications, comorbidities and mortality than type 1 diabetes mellitus. A stressful lifestyle, a shifted dietary habit and a lack of physical activity are cited as causes of early-onset T2DM. Early-onset T2DM is, therefore, an urgent public health concern requiring early prevention, efficient screening and prompt intervention. This article discusses the recent literature on the incidence, mortality, morbidity and risk variables of early-onset T2DM, and the current priorities and prospective directions.

Diabetes mellitus (DM) is a group of metabolic disorders marked by elevated blood glucose. It has many subtypes, including type 1 diabetes mellitus (T1DM), type 2 diabetes mellitus (T2DM), gestational diabetes and neonatal diabetes; of these, T1DM and T2DM are the most common. Compared with T1DM, which is characterized by a chronic lack of insulin production due to an autoimmune illness, T2DM is generally accompanied by insulin resistance.^1^ T2DM encompasses symptoms such as polyuria, polydipsia, polyphagia, blurred vision, fatigue and non-healing wounds. Obesity is the defining characteristic of T2DM, even though both type 1 and type 2 can cause weight loss.

T2DM has become a global public health crisis with far-reaching effects on people's lives. Previously thought to exclusively affect those in their middle to older age, T2DM has dramatically increased in frequency and incidence among younger people (those aged under 40 years), adolescents and even young children since the 2000s.^2^ This increase in T2DM results from increased obesity rates, and many nations now report substantial numbers of patients with early-onset T2DM. Over 85% of adolescents diagnosed with T2DM are overweight or obese.^2^Young individuals with T2DM are frequently oblivious to their progression through pre-diabetic elevated blood sugar levels.

The age-standardized incidence and disability-adjusted life-year rates of T2DM rose significantly between 1990 and 2019 (p<0.001) among adolescents and young adults worldwide: age-standardized incidence rate (per 100,000 people) increased from 117.22 in 1990 (95% confidence interval [CI] 117.07–117.36) to 183.36 in 2019 (95% CI 183.21–183.51), and the disability-adjusted life-year rate (per 100,000 people) increased from 106.34 (95% CI 106.20–106.48) to 149.61 (95% CI 149.47–149.75) from 1990 to 2019, respectively.^3^ At the same time, the average age-standardized mortality rate (per 100,000 people) was 0.77 (95% CI 0.76–0.78) in 2019, up from 0.74 (95% CI 0.72– 0.75) in 1990. According to the Centers for Disease Control and Prevention, the current prevalence of T2DM in children and adolescents is estimated to increase four-fold in the next 40 years.^1^

Because it affects the majority of the body's organs, T2DM can have extremely distressing consequences. Many people with diabetes develop irreversible vision loss from complications such as diabetic retinopathy, chronic kidney failure from diabetic nephropathy, and below-the-knee amputations from neuronal and vascular damage that leaves them restricted to wheelchairs. Non-vascular complications of diabetes – including non-alcoholic fatty liver disease, psychiatric diseases such as depressive disorders, cognitive impairment, frequent infections and disability – are on the rise. In addition to having a higher chance of developing health problems than people with T1DM, patients diagnosed with T2DM at younger ages undergo a more rapid reduction in β-cell function than those with later-onset T2DM.^1^ Therefore, early-onset T2DM is currently a more challenging clinical condition compared to late-onset T2DM.

Risk factors that contribute to early-onset T2DM include female sex, overweight and obesity, low birth weight, family history of T2DM, ethnicity and socio-economic status. Women aged under 30 years have a higher incidence of T2DM than men, perhaps due to pregnancy and polycystic ovary syndrome which are associated with insulin resistance.^3^ Additionally, dietary factors contribute to the high prevalence of diabetes. As of 2018, seven out of ten new cases of T2DM are attributable to suboptimal intakes of various dietary factors (e.g. refined rice and wheat, unprocessed red meat and fruit juice) according to universally typical and stratified estimations of dietary intake and T2DM incidence.^4^ Worldwide, excessive consumption of detrimental dietary factors generated a higher percentage of this burden (60.8%) than inadequate consumption of preventative dietary elements (39.2%).^4^

The rapid disease development witnessed in early-o nset T2DM suggests that it may differ in pathogenesis from T2DM diagnosed later in life. Current evidence suggests that obesity drives β-cell dysfunction, insulin resistance and other pathways in both early-onset and later-onset DM. However, substantial data suggests that the gradual loss of β cells in people with T2DM is much faster in younger patients than in older individuals.^2^ Obesity is more prevalent in early-onset T2DM than in later-onset T2DM. For instance, studies indicate that more than 80% of adolescents with T2DM are obese at the time of diagnosis, as opposed to adults with T2DM.^2^ In addition, the article also reveals that young individuals with T2DM appear to have three times more fat in their muscles and liver than older adults with T2DM.^2^ These results implicate obesity-related mechanisms as significant factors contributing to the development of early-onset T2DM.

Controlling T2DM involves a combination of lifestyle modifications and medication. Adopting a healthy lifestyle can reduce the risk of developing diabetes. Study shows that the relative risk reduction was 74% for those who exceeded the weight loss target of 5%, and 80% for those who exceeded the exercise goal of 4 hours per week.^2^ Avoiding highly processed foods, sugary drinks and saturated fats, eating less red and processed meat, and quitting smoking also play a key role.Unrecognized hyperglycaemia will inevitably result in potentially fatal microvascular and macrovascular injury. Therefore, effective screening for T2DM is required. Regarding the drug-based clinical treatment of T2DM in adolescents, treatment should aim to normalize haemoglobin A1c (HbA1c) and blood glucose levels, with regular examinations every 3 months by a diabetes response team, as recommended by the American Diabetes Association.^5^ It is also essential to effectively manage T2DM comorbidities, such as high blood pressure and dyslipidaemia. HbA1c levels should be measured at each visit every 3 months to achieve a level <7%. Cholesterol levels, hepatic function and sleep apnoea symptoms should be assessed during annual check-ups. Uncontrolled diabetes causes ulcer, generally in the lower extremity, which most often leads to amputation. To prevent neuropathic ulcers, appropriate footwear and foot care are essential.

Primary prevention is also essential for mitigating the detrimental consequences of diabetes on individuals and communities. Patient education has emerged as a central focus in today's healthcare systems; we can, however, gradually reduce the prevalence of early-onset diabetes through periodic public health campaigns and education in early childhood. In addition, the most prudent remedies involve resisting the problem through widespread awareness and collaboration with children, educational institutions and societies. The majority of studies on preventing adolescent obesity and early-onset diabetes have singled out the school system as the key target for intervention.^6^ Several organizations' missions centre on spreading awareness of the need to maintain a healthy lifestyle and community. Children, caregivers and the communities can acquire adequate knowledge regarding DM if a plan is standardized and maintained over time to make information more accessible through effective health campaigns; moreover, school administrators are strongly encouraged to implement it concurrently. Therefore, involving young people and their families, the educational institution, and the wider community in this prevention effort is important.
